# Acute Flaccid Paralysis Associated with Novel Enterovirus C105

**DOI:** 10.3201/eid2110.150759

**Published:** 2015-10

**Authors:** Liana M. Horner, Melinda D. Poulter, J. Nicholas Brenton, Ronald B. Turner

**Affiliations:** University of Virginia School of Medicine, Charlottesville, Virginia, USA

**Keywords:** enterovirus, rhinovirus, flaccid paralysis, viruses, enterovirus C105

## Abstract

An outbreak of acute flaccid paralysis among children in the United States during summer 2014 was tentatively associated with enterovirus D68 infection. This syndrome in a child in fall 2014 was associated with enterovirus C105 infection. The presence of this virus strain in North America may pose a diagnostic challenge.

During the summer of 2014, the Centers for Disease Control and Prevention (CDC) reported an unusual increase in the frequency of acute flaccid myelitis among children in the United States (*1*). A case definition was developed, and clinicians were urged to report new cases to CDC and state health departments. This outbreak occurred coincidentally with an outbreak of respiratory disease caused by enterovirus D68 (EV-D68) (*2*). The simultaneous occurrence of the neurologic disease and the widespread occurrence of an unusual respiratory enterovirus syndrome raised suspicion that the 2 outbreaks might be linked (*3*,*4*). We report a case that met the CDC case definition of acute flaccid myelitis but was associated with isolation of a novel enterovirus, C105, which has been previously isolated from a patient with flaccid paralysis. The presence of this virus strain in North America may contribute to the incidence of flaccid paralysis and may also pose a diagnostic challenge in clinical laboratories.

## The Case

The patient was a 6-year-old previously healthy girl examined at the University of Virginia Children’s Hospital in October 2014 for acute onset of progressive right upper extremity weakness. Within the 2 weeks before the patient’s presentation to the hospital, she and her family members had been ill with a mild cough and rhinorrhea; 4 days before presentation, the patient had experienced low-grade fever (100.4°F), frontal headache, fatigue, and intermittent pain in the right ear and right axilla. The fever lasted only 1 day; the cough, fatigue, and headache improved over the next 2 days, but the patient continued to report right arm pain. On the day before seeking care, her parents observed that she had a right shoulder droop and difficulty using her right hand. No associated visual or mental status changes; difficulty with speech, swallowing, or respiration; or bowel/bladder disturbance were noted. Physical examination detected right upper extremity weakness; absent right biceps, triceps, and brachioradialis deep tendon reflexes; and a diminished right patellar reflex. Muscle strength was more severely affected in the proximal than in the distal right upper extremity. Sensation was intact. A diffuse papular rash was noted on the patient’s back.

Magnetic resonance (MR) images of the spine revealed longitudinally extensive gray matter hyperintensity within the central cord at C3–7 and T11–12/L1 with associated edema (Figure). An MR image of the brain was unremarkable. Examination of cerebrospinal fluid (CSF) detected 5 × 10^6^ leukocytes/L (82% lymphocytes), 0.9 × 10^6^ erythrocytes/L, 3 mmol glucose/L, and 280 mg protein/L. The IgG index R-value (relative IgG/albumin ratio in CSF and serum) was mildly elevated at 0.73, but oligoclonal bands were absent. Testing of a nasopharyngeal swab specimen with the xTAG Respiratory Viral Panel (Luminex, Austin, TX, USA) produced positive results for picornavirus. PCR for enterovirus in CSF, performed by using the XpertEV test (Cepheid, Sunnyvale, CA, USA) was negative. Subsequent confirmatory testing at the Division of Consolidated Laboratories for the Commonwealth of Virginia also produced negative results. Subsequent testing of a nasopharyngeal swab specimen by sequence analysis of the viral protein 1 capsid gene revealed the presence of enterovirus C105 (*5*). Culture of a fecal specimen produced negative results for enterovirus. Other negative results were obtained from serologic testing for *Borrelia burgdorferi*, arboviruses, and neuromyelitis optica IgG.

**Figure:**
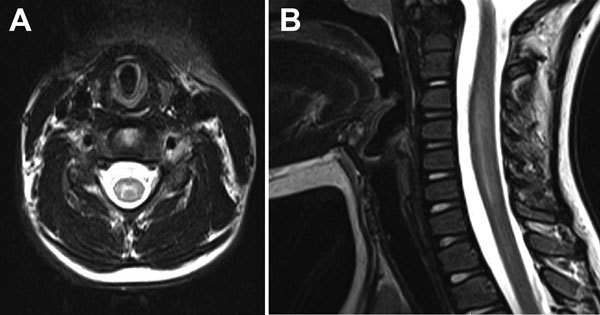
Magnetic resonance imaging of 6-year-old girl with flaccid paralysis and enterovirus C106 infection, Virginia, USA, October 2014. A) Axial T2-weighted image of the cervical spine demonstrating abnormal hyperintensity of the central gray matter (right to left). B) Sagittal T2-weighted image of the cervical spinal cord demonstrating faint longitudinally extensive central hyperintensity and associated cord edema.

The patient received intravenous immunoglobulin, 2 g/kg divided daily over 5 days, but did not respond. She continued to report intermittent pain in her right arm, low-back pain, and bilateral thigh pain when walking. During her hospital stay, the patient’s weakness remained stable (no substantial progression or improvement). After discharge, her pain spontaneously resolved, and 8 months after illness onset (most recent follow-up visit), her proximal right arm weakness improved and the strength in her distal right arm had almost resolved.

## Conclusions

Enterovirus C105 was first detected in 2010 in patients from Peru and the Republic of Congo; subsequent reports suggest that the virus is circulating worldwide (*6*–*9*). The enterovirus C species includes the polioviruses and 11 enterovirus serotypes previously classified as coxsackie A viruses. Enterovirus C105 seems to be a member of a recently detected subspecies that is distinguished from the other enterovirus C viruses by a divergent 5′-untranslated region (UTR) (*10*). The clinical spectrum associated with these recent isolates is still poorly defined. Most isolates have been associated with respiratory syndromes, but the isolate from the Republic of Congo was associated with fatal acute flaccid paralysis. 

Detecting the virus in clinical specimens may be challenging because of the divergence of the 5′-UTR in members of this newly emerging subspecies of enterovirus C (*10*,*11*). Many broad-specificity enterovirus real-time reverse transcription PCRs target conserved regions of the 5′-UTR. The sequence divergence in the subspecies occurs in regions that are targeted by these diagnostic assays and may interfere with recognition of the virus by the primers. For the patient we report, virus in the nasopharyngeal swab sample was identified as a picornavirus by the xTAG Respiratory Virus Panel and by sequence analysis of the viral protein 1 capsid gene (*5*).

In the United States, the outbreak of acute flaccid myelitis that began in the summer of 2014 affected 118 children (http://www.cdc.gov/ncird/investigation/viral/sep2014/investigation.html). The case definition for the outbreak is acute onset of focal limb weakness associated with a spinal cord lesion restricted to the gray matter (according to MR images) in a child <21 years of age. The patient reported here met this case definition. A detailed report of 88 of these children noted that most (81%) had experienced a preceding respiratory illness, similar to that described by the patient reported here (*12*). Despite the suspicions that EV-D68 may be a cause of the neurologic syndrome (*3*,*4*), early reports indicate that EV-D68 was detected in only 8 (20%) of 41 of the flaccid myelitis patients tested, and no enterovirus has been detected in spinal fluid (*12*). EV-D68 was not detected in specimens from the patient reported here, and EV-D68 was not epidemic in central Virginia. However, we did not have access to convalescent serum samples for antibody testing, and it is not possible to definitively exclude the possibility of an undetected co-infection.

EV-D68 is unusual in that, although it is a member of the enterovirus D species, this virus has phenotypic characteristics that are more consistent with rhinoviruses. The virus is acid labile and preferentially grows at 33°C, characteristics that have been used to classify picornaviruses as rhinoviruses (*13*). The virus also seems to be similar to rhinoviruses in its propensity to cause an afebrile respiratory syndrome and to exacerbate asthma symptoms in asthma patients (*2*). Rhinoviruses are not associated with systemic disease, and virus replication is limited to the respiratory tract, a characteristic that has been attributed to the temperature sensitivity of the virus. Although cases of flaccid paralysis associated with isolation of EV-D68 from spinal fluid have been reported, the role of EV-D68 in the current outbreak remains to be determined. As the results from this case indicate, it is possible that other viral pathogens with neurovirulence may be contributing to the outbreak. 
